# Supplementary material for: The utility of measures of anterior segment parameters of a Pentacam Scheimpflug tomographer in discriminating high myopic astigmatism from keratoconus
Source: PLoS One. 2021 Dec 2;16(12):e0260648. doi: 10.1371/journal.pone.0260648 (PMC8638937; doi:10.1371/journal.pone.0260648)
Supplement: Attachment — Submitted filename: Response to Reviewers 2.docx [file pone.0260648.s002.docx]

| **Dear Editor**  **RESPONSE TO REVIEWERS.**  **RE:** **The utility of measures of anterior segment parameters of pentacam scheimpflug tomographer in discriminating high myopic astigmatism from keratoconus** **(PONE-D-21-20839R1)** | | | |
| --- | --- | --- | --- |
| Reviewer | Original comment by the reviewer | Reply by the author(s) | Changes on page number |
| Academic Editor | 1. Thank you for updating your data availability statement. You note that your data are available within the Supporting Information files, but no such files have been included with your submission. At this time we ask that you please upload your minimal data set as a Supporting Information file, or to a public repository such as Figshare or Dryad.  Please also ensure that when you upload your file you include separate captions for your supplementary files at the end of your manuscript. As soon as you confirm the location of the data underlying your findings, we will be able to proceed with the review of your submission. | Thank you. The manuscript has uploaded. | Please see the uploaded files. |
|  | 2. Please ensure that you refer to Table 4 and 5 in your text as, if accepted, production will need this reference to link the reader to the Table. | Please we have referred to the table in the text. Thank you so much for drawing out attention. | See red highlights of the table heading. |
